# The Restriction of Zoonotic PERV Transmission by Human APOBEC3G

**DOI:** 10.1371/journal.pone.0000893

**Published:** 2007-09-12

**Authors:** Stefán R. Jónsson, Rebecca S. LaRue, Mark D. Stenglein, Scott C. Fahrenkrug, Valgerdur Andrésdóttir, Reuben S. Harris

**Affiliations:** 1 Department of Biochemistry, Molecular Biology and Biophysics, University of Minnesota, Minneapolis, Minnesota, United States of America; 2 Institute for Molecular Virology, University of Minnesota, Minneapolis, Minnesota, United States of America; 3 Arnold and Mabel Beckman Center for Transposon Research, University of Minnesota, Minneapolis, Minnesota, United States of America; 4 Institute for Experimental Pathology, University of Iceland, Reykjavík, Iceland; 5 Department of Animal Sciences, University of Minnesota, St. Paul, Minnesota, United States of America; University of California at San Francisco, United States of America

## Abstract

The human APOBEC3G protein is an innate anti-viral factor that can dominantly inhibit the replication of some endogenous and exogenous retroviruses. The prospects of purposefully harnessing such an anti-viral defense are under investigation. Here, long-term co-culture experiments were used to show that porcine endogenous retrovirus (PERV) transmission from pig to human cells is reduced to nearly undetectable levels by expressing human APOBEC3G in virus-producing pig kidney cells. Inhibition occurred by a deamination-independent mechanism, likely after particle production but before the virus could immortalize by integration into human genomic DNA. PERV inhibition did not require the DNA cytosine deaminase activity of APOBEC3G and, correspondingly, APOBEC3G-attributable hypermutations were not detected. In contrast, over-expression of the sole endogenous APOBEC3 protein of pigs failed to interfere significantly with PERV transmission. Together, these data constitute the first proof-of-principle demonstration that APOBEC3 proteins can be used to fortify the innate anti-viral defenses of cells to prevent the zoonotic transmission of an endogenous retrovirus. These studies suggest that human APOBEC3G-transgenic pigs will provide safer, PERV-less xenotransplantation resources and that analogous cross-species APOBEC3-dependent restriction strategies may be useful for thwarting other endogenous as well as exogenous retrovirus infections.

## Introduction

Viral zoonoses have impacted human populations through the ages. The 1918 influenza epidemic, the 2003 SARS incident and the ongoing HIV/AIDS pandemic constitute clear examples of virus transfer from birds [1], bats [2] and chimpanzees [3]. Domesticated animals can also function as zoonotic intermediates (*e.g*., [4], [5]). Additional and unprecedented opportunities for zoonoses occur when live cells, tissues or organs are transplanted from one species to another [6]. However, despite risks and technical and immunological challenges, several xenotransplantation procedures have shown preclinical promise for treating diabetes, heart, kidney and other human diseases (*e.g*., [7]–[9]).

Pigs are favourable xenotransplantation sources because of their human-like physiology, large litters, short gestation period and genetic malleability [10]. However, pig to human virus transmission has been a concern since it was shown that porcine endogenous retroviruses (PERVs) could infect human cells in culture [6], [11]–[13]. Although PERV transmission has yet to be documented in xenotransplantation patients, significant concerns still exist regarding PERV and other potentially pathogenic viruses [14], [15]. Strategies to reduce the likelihood of PERV transmission have been proposed, such as selective breeding for lower levels of PERV, RNAi transgenesis to knock-down PERV expression or systematic deletion of active PERV copies (*e.g*., [15], [16]). The first two are unlikely to be completely effective or risk-free and the third, albeit theoretically feasible, may be overly technical and prohibitively expensive. Therefore, alternative, robust and cost-effective methods to reduce PERV transmission and possible xenozoonotic infections are desirable.

APOBEC3G is a single-strand DNA cytosine deaminase best understood as a potent inhibitor of HIV-1 replication [17]–[23]. It can however also inhibit a variety of other exogenous and endogenous retroviruses/elements (*e.g*., [17], [18], [24]–[29]). APOBEC3G engages an assembling retrovirus particle, accesses the RNA genome-containing virus core and, upon reverse transcription, deaminates cDNA cytosines to uracils (C-to-U). Catastrophic levels of uracil either directly inactivate the coding capacity of the virus or trigger the degradation of the viral DNA. The former manifests as genomic strand-specific guanine to adenine (G-to-A) hypermutations (cDNA strand C-to-T transitions). However, in several instances, it is noteworthy that the deaminase activity of APOBEC3G or other APOBEC3 proteins is partly or even completely dispensable (*e.g*., HIV-1, hepatitis B virus, L1 and Alu [27]–[32]).

Interestingly, throughout evolution, the retroviruses of many mammals appear to have become largely immune to APOBEC3G or to the APOBEC3G-like proteins of their hosts. HIV-1 expresses an accessory protein, Vif, which neutralizes APOBEC3G through ubiquitination and degradation [33]–[36]. Simian immunodeficiency virus (SIV) uses a similar Vif-dependent mechanism [17], [26]. Foamy viruses employ an unrelated viral protein called Bet, for which the precise neutralization mechanism is currently unclear [37], [38]. Murine leukaemia virus (MLV) and human T-lymphotrophic virus-1 (HTLV-1) may simply avoid APOBEC3 proteins by preventing encapsidation [26], [39], [40].

However, cell-based studies have indicated that an APOBEC3 protein from a mammal to which the virus has not yet adapted may provide an effective strategy for thwarting species-specific viral counter-defenses. For instance, human APOBEC3G can potently inhibit the replication of SIV (except isolates such as SIV_cpz_ which encode a Vif protein closely related to that of HIV-1), feline foamy virus and MLV (*e.g*., [17]–[20], [25], [26], [39], [41], [42]). Similarly, mouse APOBEC3 can potently block HIV-1 replication (regardless of Vif), although it is completely unable to impede the replication of MLV [26], [41]. One of the most dramatic examples to date used human and mouse APOBEC3 proteins to inhibit the mobilization of the yeast retrotransposons Ty1 and Ty2 [43], [44]. Thus, it is reasonable to hypothesize that cross-species expression of an APOBEC3 protein may be used to create a powerful barrier to impede or perhaps even block retrovirus infection. Here, this rationale is applied to the specific question of whether human APOBEC3G expression can inhibit the transmission of PERV from pig to human cells. The results demonstrate that PERV transmission can be strongly inhibited by APOBEC3G.

## Results

### A Co-culture Assay to Monitor PERV Transmission

**Figure 1:**
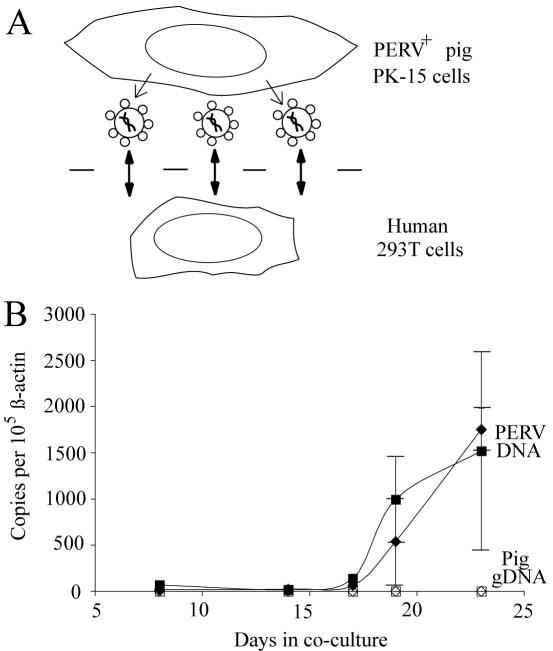
PERV Transmission Assay. (A) Schematic of the co-culture system. PERV transmitting PK-15 cells are grown on top of the membrane of the insert and human 293T cells on the bottom of the well of the culture dish. Virus particles are depicted diffusing through membrane pores. (B) The zoonotic transmission of PERV from PK-15 cells to 293T cells is shown by the time-dependent accumulation of PERV *pol* gene DNA in the human cells (solid diamonds and squares). No concomitant transfer of pig genomic DNA occurred through the duration of these experiments (open diamonds and squares). This graph summarizes data for two independently derived PK-15 clones, V1 (squares) and V2 (diamonds). All data points were calculated using results from duplicate Q-PCR reactions of genomic DNA from three parallel (but independent) co-cultures. The error bars indicate the SEM. See the Materials and Methods and Online Figure S1 for additional details, representative raw data and controls.

To determine whether expression of human APOBEC3G would inhibit the transfer of PERV from pig to human cells it was first necessary to establish a long-term co-culture system. A trans-well assay was set up to monitor PERV transmission from pig kidney PK-15 fibroblasts to recipient human embryonic kidney 293T cells (Figure 1A). These two cell types were used because transmission from PK-15 to 293 cells had been reported previously [11]. The trans-well system enabled co-cultures to be sustained for several weeks, and it facilitated the recovery of each cell type for downstream analyses. An additional benefit of this co-culture system (not provided by transient assays) is that it enables the simultaneous analysis of multiple, endogenous PERV elements, which are precisely the targets one would want to monitor and ideally inhibit in (xeno)transplantation procedures.

At each co-culture passage point, surplus human 293T cells were used to prepare genomic DNA. PERV transmission was monitored by subjecting these samples to quantitative (Q)-PCR. PERV-specific *pol* gene PCR products could be detected in the human genomic DNA samples after approximately two weeks of continuous co-culture (Figure 1B). From the point of first detection onward, the total number of PERV transmissions continued to increase, averaging 190 new events per day per 50,000 cells (10^5^ *beta-actin* copies; SEM = 62; n = 5 experiments). Importantly, pig cells did not breach the 293T cell compartment because Q-PCR analyses of the same human genomic DNA samples failed to detect a concomitant transfer of pig genomic DNA (Figure 1B; also see Online Figure S1 for PERV *pol* gene Q-PCR standard curves, representative PERV-specific datasets and human *beta-actin* controls). Moreover, PERV copy number did not increase over a two week interval when infected 293T cells were grown in isolation, indicating that PERV was not replicating in the human cells and that the majority of the observed transmission events were derived from the PK-15 cells (*i.e*., new events). These results combine to indicate that the trans-well assay provided a robust system for monitoring *bona fide* zoonotic PERV transmissions.

### Human APOBEC3G Inhibits PERV Transmission

**Figure 2:**
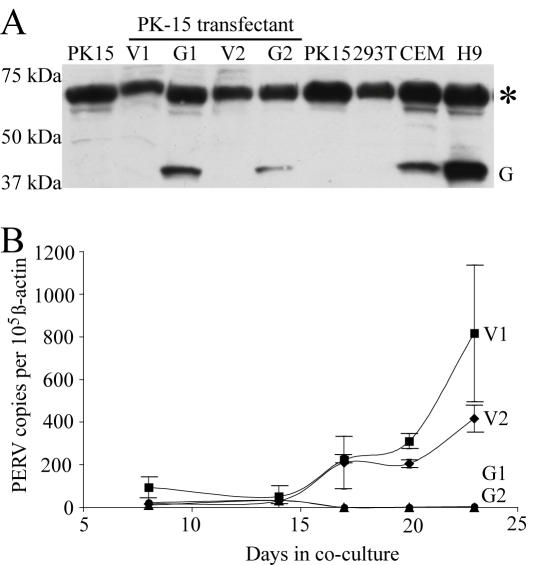
Human APOBEC3G Inhibits PERV Transmission. (A) An immunoblot showing PK-15 clones expressing human APOBEC3G (G1 and G2) or a control vector (V1 and V2). PK-15 and 293T cell lysates were used as negative controls. CEM and H9 were used as positive controls for APOBEC3G expression. A non-specific (but pan-species) band is shown as a protein loading control (marked by an asterisk). (B) Q-PCR data using genomic DNA prepared from 293T cells co-cultured with two independently derived APOBEC3G expressing PK-15 clones (G1 and G2, circles and triangles, respectively) or two vector control clones (V1 and V2, diamonds and squares, respectively). The experimental parameters are identical to those used in Figure 1B.

The second key step in addressing our experimental question was isolating PK-15 clones that stably expressed human APOBEC3G. Clones expressing human APOBEC3G cDNA or an empty vector control were established in parallel (Figure 2A). Immunoblotting identified clones with APOBEC3G levels similar to those in known APOBEC3G-expressing human T cell lines CEM and H9, which are non-permissive for growth of Vif-deficient HIV-1 [19]. Although it was impossible to achieve a physiologic expression level, the comparative immunoblot at least ensured that the levels of APOBEC3G were equal or lower than those present in well-studied, non-permissive human T cell lines.

Co-culture experiments were set up to compare PERV transmission from two independently derived PK-15 clones expressing human APOBEC3G and two vector expressing controls. Remarkably, the human APOBEC3G expressing PK-15 clones showed levels of PERV transmission that were lower than the Q-PCR detection threshold of approximately 10 copies (Figure 2B; Online Figure S1). In contrast, the control clones showed high levels of PERV transfer by co-culture day 17 and transmission events continued to accumulate through the duration of the experiment. The kinetics of PERV transmission were similar to those reported in Figure 1B (these results also contributed to the aforementioned transfer rate calculations). These data were further corroborated by additional experiments where PERV transmission was monitored simultaneously by Q-PCR and by reverse-transcriptase ELISA assays (Online Figure S2).

### The Sub-cellular Distribution of Human APOBEC3G Is Virtually Identical in Human and Pig Cell Lines

**Figure 3:**
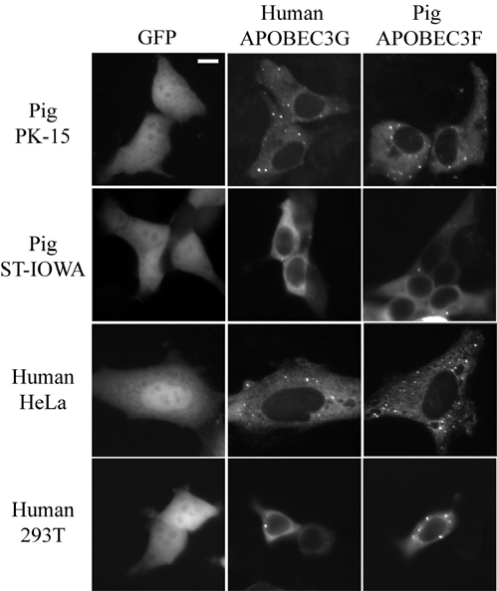
The Sub-cellular Distribution of Human APOBEC3G in Human and Pig Cell Lines. Sub-cellular distributions of GFP, human APOBEC3G-GFP and pig APOBEC3F-GFP in the indicated live pig and human cell lines. The scale bar in the top left panel indicates 10 µM.

An ultimate application of the technology described here raises the potential problem that human APOBEC3G may not be subjected to (proper) post-translational regulation in pig cells and it may therefore promote carcinogenesis. Expression of human APOBEC3G in a heterologous system has been shown to trigger elevated levels of genomic C/G-to-T/A transition mutation [44]. Therefore, to help mitigate this risk (in addition to establishing clones that expressed relatively modest APOBEC3G levels; above), we asked whether the predominantly cytoplasmic localization pattern of human APOBEC3G would be maintained in PK-15 and in a swine testes cell line, ST-IOWA (Figure 3; compare with other APOBEC3G reports [18], [35], [41]). Unlike some other APOBEC3 proteins such as human APOBEC3B, which is mostly nuclear, both human APOBEC3G and pig APOBEC3F appeared predominantly cytoplasmic in either the human or the pig cell lines (Figure 3; [28], [31], [41]). Both proteins also appeared to concentrate in cytoplasmic punctae, which varied in number and were apparent in some of the cells regardless of species of origin (described previously for APOBEC3G; *e.g*., [45], [46]). Overall, these near-identical localization patterns suggested that human APOBEC3G is not aberrantly regulated in pig cells and, interestingly, that these proteins might be subjected to the same cellular regulatory mechanism(s).

### PERV Appears Resistant to Porcine APOBEC3F

During the course of these experiments, we reported some of the activities of pig APOBEC3F [41]. It could strongly inhibit the replication of HIV (regardless of Vif) and modestly inhibit the replication of MLV, a gamma-retrovirus phylogenetically related to PERV. Therefore, we wondered whether pig APOBEC3F was expressed in PK-15 and, if so, whether PERV resists this cellular defense. To begin to address this possibility, RT-PCR was used to test PK-15 cells for pig APOBEC3F expression. Pig *APOBEC3F* mRNA was detected readily (Online Figure S3A). Full cDNA sequencing revealed that the predicted APOBEC3F protein of PK-15 cells was 98% identical to the variant we reported previously [41]. Eight amino acid differences were found, but both the PK15 and the previously reported APOBEC3F sequences were represented in pig genomic DNA sequences suggesting that these may be breed-specific polymorphisms (R.S.L. and R.S.H., manuscript in preparation). These observations indicated that either PERV resists the endogenous APOBEC3F protein of its host or that the level of suppression by pig APOBEC3F is not sufficient to inhibit PERV transmission.

To begin to distinguish between these two hypotheses, PK-15 clones over-expressing pig APOBEC3F were established and used in transmission experiments. The former hypothesis was favored because pig APOBEC3F over-expression did not significantly interfere with PERV transmission (Online Figure S3B). These results were further supported by PCR experiments showing that PERV could be amplified readily from 293T cells that had been co-cultured with PK-15 over-expressing pig APOBEC3F (unlike the APOBEC3G scenario; below).

We further noted that it is highly unlikely that another resident APOBEC3 protein contributes to PERV restriction, because genomic DNA sequencing showed that pigs have only one *APOBEC3* gene, *APOBEC3F* (R.S.L. and R.S.H., manuscript in preparation). These observations combined to indicate that PERV is resistant to the endogenous APOBEC3 protein of its host. In hindsight, this was not particularly surprising given the emerging trend that (successful) retroviruses are selected in part by their ability to evade the APOBEC3 proteins of their host species (see Introduction).

### Evidence that Human APOBEC3G Inhibits PERV By a Deamination-Independent Mechanism

The hallmark of APOBEC3G-dependent retrovirus restriction is plus-strand G-to-A hypermutation, which is caused by the deamination of minus-strand cDNA C-to-U during reverse transcription [17], [18], [20], [24], [25]. The deamination of cytosines within single-strand DNA requires glutamate 259 (E259) of APOBEC3G [47]–[49]. Based on homology to structurally defined deaminases, E259 likely functions by helping position the water molecule that ultimately initiates the deamination reaction by attacking the cytosine ring (as a hydroxide; reviewed by [17], [23], [50], [51]).

**Figure 4:**
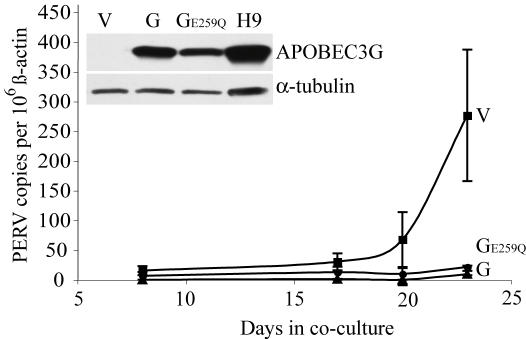
The APOBEC3G-dependent Inhibition of PERV Transmission Is Deamination-Independent. PERV-specific Q-PCR data using genomic DNA prepared from 293T cells co-cultured with PK-15 clones expressing APOBEC3G (G; triangles), APOBEC3G-E259Q (GE259Q; circles) or empty vector (V; squares). Two datasets, each with an independent PK-15 clone in three replica co-culture wells, were collected in parallel and averaged for each data point. One standard error of the mean is shown. The experimental parameters are identical to those used in Figure 1B. The inset immunoblots show the APOBEC3G and α-tubulin levels of representative PK-15 clones expressing the indicated construct. The human T cell line H9 provided a positive control for APOBEC3G expression.

To determine whether DNA deamination is required the APOBEC3G-dependent inhibition of PERV transmission, we established a new set of PK-15 clones expressing APOBEC3G, APOBEC3G E259Q or a vector control (Figure 4, inset). Surprisingly, both APOBEC3G and the E259Q derivative diminished PERV transmission to near background levels (Figure 4). These data demonstrated that the mechanism of inhibition does not require the DNA deaminase activity of APOBEC3G. These data were further supported by the fact that plus strand G-to-A hypermutations were not apparent in the DNA of the rare transmission events that occurred in the presence of human APOBEC3G (below).

### Genetic Variation in Zoonosed PERV DNA Sequences

**Figure 5:**
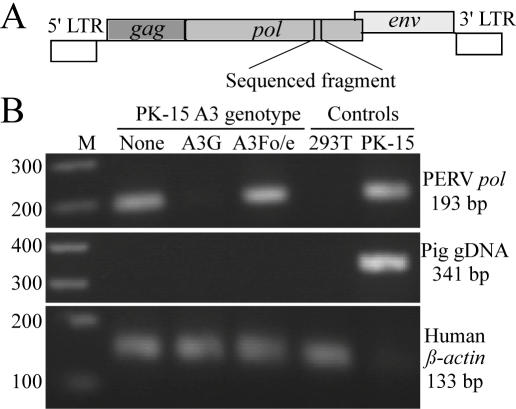
Genetic Variation in Zoonosed PERV DNA Sequences. (A) A schematic of the PERV genome showing coding regions (*gag*, *pol* and *env*) and long-terminal repeats (LTRs). The relevant 193 bp *pol* gene fragment is indicated. (B) An ethidium bromide-stained agarose gel showing that PERV *pol* gene DNA was amplified readily from 293T cells cultured with PK-15 control clones (None) and pig APOBEC3F over-expressing clones (A3Fo/e) but not with PK-15 clones expressing human APOBEC3G (A3G; top panel). PERV *pol* gene DNA (top panel) and pig genomic DNA (*APOBEC3F* locus; middle panel) PCR products were detected in PK-15 genomic DNA, whereas human *beta-actin* was strongly detected in the 293T cell genomic DNA samples (a much weaker amplification of pig *beta-actin* occurred because the human primers had partial complementarity to pig sequences, 20/21 and 17/18 nucleotides).

To begin to genotype the infectious PERVs and to further probe the mechanism of PERV restriction by human APOBEC3G, the PERV *pol* gene DNA was amplified from human 293T cells, cloned and sequenced (Figure 5A; Online Figure S4). Twenty-nine and twenty-two sequences were analysed from APOBEC3G and control experiments, respectively. To minimize possible PCR biases, any sequence that was recovered multiple times was considered one event, unless it arose from independent experiments. These DNA sequence analyses revealed several important points.

First, in contrast to vector control and pig APOBEC3F over-expressing co-cultures, PERV *pol* gene DNA was difficult to amplify from the genomic DNA of 293T cells that had been co-cultured with APOBEC3G-expressing PK-15 cells (Figure 5B and every significant sampling point in our Q-PCR experiments). Taking this together with the observation that APOBEC3G does not effect PK-15 virus production (similar RT levels were observed in cell-free supernatants in the presence or absence of APOBEC3G; data not shown), we infer that APOBEC3G restricts PERV transmission after virus production but before provirus integration (*i.e*., between entry and integration). APOBEC3G may restrict PERV at an early reverse transcription stage, possibly by interfering with primer binding, DNA synthesis and/or integration as shown recently for APOBEC3G and HIV-1 substrates (*e.g*., [30], [52]–[55]).

Second, control co-culture PERV transmission events were exceptionally diverse, as 11 unique *pol* sequences were detected and only 4 were found multiple times (Online Figure S4). These data suggested that PK-15 cells have at least 11 active PERVs capable of infecting human 293T cells, a number consistent with previous studies that reported the existence of approximately 17–50 PERV copies in total (with only a fraction being replication-competent; [11], [12]). In contrast, the rare PERV sequences derived from the APOBEC3G co-culture experiments showed a much lower genetic complexity. Only three unique sequences were recovered, each differing by a single nucleotide (Online Figure S4). In parallel experiments with HIV-based viruses, this APOBEC3G expression construct caused approximately 30 G-to-A hypermutations per 1000 bases analyzed (*e.g*., [41]). Thus, approximately 12 G-to-A transitions should have been recovered in these PERV DNA analyses (nearly 90 if multiply recovered sequences would have been considered). The absence of hypermutated PERV proviral DNA provided further support for a deaminase-independent mechanism of restriction, which may share features with other instances described previously (*e.g*., [27]–[30]).

## Discussion

We have established a quantitative assay to monitor the zoonotic transmission of PERV to human 293T cells. Expression of human APOBEC3G in the pig PK-15 cell line strongly inhibited PERV zoonoses, while the endogenous APOBEC3F protein of pigs appeared considerably less effective. These data are the first to show that human APOBEC3G can inhibit PERV and the first to demonstrate that APOBEC3 proteins can be used purposefully to reduce if not prevent zoonotic retroviral infections. These results were not anticipated because human APOBEC3G has a relatively weak effect against the PERV-related gamma-retrovirus MLV [26], [39].

Our data indicate that the engineering of pigs to express human APOBEC3G (or an equally potent non-porcine APOBEC3) may result in animals whose cells and tissues are much less likely to disseminate functional PERV. The deamination-independence of the restriction mechanism suggests that a catalytically inert APOBEC3G protein, such as E259Q, may be equally potent and simultaneously reduce the risk of cancer-promoting mutagenesis. APOBEC3G or APOBEC3G-E295Q expressing pigs may therefore constitute safer source animals for pig-to-human xenotransplantation procedures. In contrast to knockdown, knockout (by gene targeting or selective breeding) or most chemical-based anti-viral approaches to neutralize PERV [15], the APOBEC3 anti-viral defense system has several advantages including a potentially broad neutralizing activity (effective against PERV and likely several other endogenous and exogenous viruses) and an applicability to situations where many copies of a virus are already present in a genome. Analogous transgenic applications can be envisaged, such as using cross-species APOBEC3 expression to purposefully impede known viruses (*e.g*., the AIDS virus HIV-1 or the Hepatitis B virus HBV). Moreover, for humans and other mammals with multiple APOBEC3 proteins, our data encourage the development of methods to induce/up-regulate endogenous APOBEC3 proteins, which have the capacity but may not normally restrict a particular virus (*e.g*., human APOBEC3B and HIV-1).

## Materials and Methods

### Cell lines, plasmids and co-culture experiments

The porcine kidney PK-15 fibroblast cell line and the swine testes ST-IOWA cell line were obtained from the ATCC and cultured in Dulbecco's modified Eagle's medium (Invitrogen) supplemented with 10% fetal bovine serum (Gemini), and 25 units/ml penicillin and 25 µg/ml streptomycin at 37°C and 5% CO_2_. Human embryonic kidney 293T and HeLa (A. Bielinsky, University of Minnesota) cell lines were grown under the same conditions. The T cell lines H9 and CEM (M. Malim, Kings College London) were cultured in RPMI-1640 supplemented with 10% fetal bovine serum (Gemini), and 25 units/ml penicillin and 25 µg/ml streptomycin at 37 °C and 5% CO_2_. Plasmids encoding human APOBEC3G, human APOBEC3G-E259Q and porcine APOBEC3F were described previously [41]. The human APOBEC3G and porcine APOBEC3F cDNA sequences used here are identical to GenBank accession numbers, NM_021822 and NM_001097446, respectively.

Stable APOBEC3G- or vector control-expressing PK-15 cell lines were constructed by transfection using FuGENE6 according to the manufacturer's protocol (Roche) or by electroporation (BioRad, 250V, 950 µFa). Clones were selected using growth medium containing 1 mg/ml G418 (Roche), and APOBEC3G expressing clones were identified by immunoblotting using a polyclonal antibody toward human APOBEC3G (J. Lingappa, University of Washington). All PK-15 clones were maintained in growth medium supplemented with 250 µg/ml G418 to ensure stable expression.

Long-term co-culture assays were performed in 6 well tissue culture plates with inserts (Transwell®, Corning Inc.). This system uses a membrane with 0.4 µM diameter pores, which keeps the two cell types separated physically but simultaneously allows diffusion of nutrients and small molecules including virus particles of approximately 0.1 µM (including PERV). Each experiment was initiated with 75,000 PK-15 cells (insert) and 75,000 293T cells (well) as illustrated (Figure 1A). At 72 hr intervals, each cell type was washed with PBS, subjected to mild trypsinization and diluted into 4 parts fresh growth medium. Excess 293T cells were used to prepare genomic DNA (Qiagen DNeasy kit).

The rate of PERV transfer was calculated using the *pol* gene levels from the last two data points (usually spanning a 3 day period). The difference between these levels represents PERV *pol* gene DNA that has accumulated per 100,000 human *beta-actin* gene copies (50,000 cells assuming that the 293T cell line has two *beta-actin* copies) per time period. Individual rates from 5 independent experiments were averaged to determine the overall transmission rate (190+/−62 events per day per 50,000 cells). Data from Figures 1B, 2B and S2A contributed to rate calculations.

### Genomic DNA Q-PCR assays

Genomic DNA was isolated from human 293T cells using the DNeasy kit (Qiagen). Duplicate 25 µl PCR reactions consisting of 10 ng of 293T genomic DNA, 100 nM primers and 2× iQ SYBR Green super mix (BioRad) were run on an iCycler iQ Multicolor Real-Time PCR detection System (BioRad). The thermocycler conditions consisted of an initial denaturation of 95°C for 5 min and 50 cycles of denaturation (95°C for 15 sec) and annealing (58°C for 30 sec). After the 50 cycles, a melting curve analysis (55°C to 95°C) was performed to confirm product specificity. The cycle threshold (C_T_) was generated using BioRad software and it was used to calculate the amount of target DNA (PERV *pol* or human *beta-actin*). A standard curve was generated using the method of Dorak [56] and a dilution series (10 to 10^7^ copies) of a linearized plasmid containing the relevant 193 bp PERV *pol* gene fragment. The equation generated from the standard curve (slope and y intercept) was used to determine the efficiency of the PCR reaction and to quantify the number of PERV *pol* gene or human *beta-actin* copies in the Q-PCR reactions. PERV copy numbers were normalized to those of *beta-actin* using the method of [56]. The primer sets used in this study were: PERV *pol* (193 bp): 5′-AAC CCT TTA CCC TTT ATG TGG AT and 5′-AAA GTC AAT TTG TCA GCG TCC TT; human *beta-actin* (133 bp)[57]: 5′-ATC ATG TTT GAG ACC TTC AA and 5′-AGA TGG GCA CAG TGT GGG T; pig genomic DNA (341 bp product specific to intron 5 of the pig *APOBEC3F* locus): 5′-TGG GGA GTG TGG AAT TAA CG and 5′-GGG GGT TAA GAA CCC AAC AT.

### RT-PCR experiments

Standard reverse transcription (RT)-PCR reactions were performed using RNA prepared from PK-15 cells (TRIzol protocol, Invitrogen), M-MLV reverse transcriptase was used for cDNA synthesis using an oligo dT primer (Ambion) and Taq polymerase was used for PCR (Roche). The primers specific to pig APOBEC3F were 5′-TGG TCA CAG AGC TGA AGC AG and 5′-TTG TTT TGG AAG CAG CCT TT (175 bp). The semi-nested primer set used to detect plasmid-expressed pig APOBEC3F was 5′-CCA AGG AGC TGG TTG ATT TC (exon 6, reaction 1), 5′-CTG GAG CAA TAC AGC GAG AG (exon 7, reaction 2) and 5′-TAG AAG GCA CAG TCG AGG, with the latter being vector specific (319 bp and 190 bp products, respectively). The mammalian *beta-actin* primers were 5′-CCT TCA ATT CCA TCA TGA AGT G and 5′-CCA CAT CTG CTG GAA GGT (236 bp). These primers amplify equally well a 236 bp *beta-actin* fragment from all mammals tested, including pigs and humans (*e.g*., Online Figure S3).

### Fluorescent microscopy

The human APOBEC3G-GFP, pig APOBEC3F-GFP and GFP expression constructs were described previously [28], [41]. The pig and human cell lines were maintained as above. One day prior to transfection, 5,000–20,000 cells were seeded onto LabTek chambered coverglasses (Nunc). After 24 hrs incubation, these cells were transfected with 250 ng of the relevant plasmid construct. After 24 hrs of additional incubation, images of the live cells were acquired using a Zeiss Axiovert 200 microscope at 400× total magnification. Images were analyzed using Image J software (http://rsb.info.nih.gov/ij).

### Reverse transcriptase activity assays

Whole cell protein extracts were prepared from 293T cells by suspending 500,000 cells in PBS, sonicating twice for 5 seconds and clarifying the lysates by centrifugation. Soluble protein levels were quantified using a BioRad Bradford assay. 10 µg of cell lysate was tested for reverse transcriptase activity using a C-type-RT activity assay (Cavidi Tech) following the manufacturers' instructions. Cell-free PK-15 supernatants (PERV-containing) were assayed directly using the Cavidi Tech ELISA assay.

### PERV *pol* gene DNA sequence analyses

Human 293T cell genomic DNA was prepared from terminal co-cultures and 50 ng was used for high fidelity, PERV *pol* gene-specific PCR reactions (Phusion polymerase; Finnzymes). 193 bp products were cloned using the Zero Blunt TOPO PCR Cloning kit (Invitrogen) and sequenced (University of Minnesota Advanced Genetic Analysis Facility). Sequence comparisons were performed using Sequencher software (Gene Codes Corp.) and publicly available Clustal W alignment algorithms (http://align.genome.jp/).

## Supporting Information

Figure S1 Quantitative Real-time PCR Analyses. (A) Standard curves depicting Q-PCR data obtained using dilutions of a linearized PERV *pol* gene plasmid alone (squares) or diluted plasmid plus 10 ng of 293T cell genomic DNA (diamonds). Under both conditions, all template amounts (10 to 10^7^ copies) amplified efficiently (the log-linear slope equations are shown). The correlation co-efficiency value (R^2^), which reports the technical accuracy of the assay, is also indicated. The standard curve data points were the average of 2 independent reactions with deviations smaller than the symbols (i.e., C^T^ errors for each point ranged from 0 to 0.4). (B) Two representative control Q-PCR datasets showing the amplification of PERV *pol* gene DNA from pig PK-15 cell genomic DNA (circles). Two additional control Q-PCR datasets showing that the PERV-specific primers fail to amplify product from uninfected human 293T cell genomic DNA (squares). The reaction threshold, 10 times the mean standard deviation of the background fluorescence level (BioRad), is indicated. (C) Representative co-culture Q-PCR amplification curves of PERV *pol* gene DNA. Template genomic DNA isolated from human 293T cells co-cultured with vector expressing PK-15 cells (diamonds) or human APOBEC3G-expressing PK-15 cells (triangles) was used. (D) Representative Q-PCR amplification curves of the 293T cell *beta-actin* gene, which served as an internal standard for quantifying the real-time PCR data. Raw Q-PCR data will be made available on request.
(9.93 MB TIF)

Figure S2 APOBEC3G inhibits PERV transmission. (A) A graph showing the accumulation of PERV *pol* gene-specific PCR products in 293T cells co-cultured with a control cell line (V3) but not with an APOBEC3G-expressing cell line (G1). The data points were an average of two Q-PCR runs and the difference between each run was smaller than the plotted symbol. The experimental parameters were identical to those used in the experiments shown in Figures 1B and 2B. (B) Relative levels of reverse transcriptase(RT)-activity detected in soluble extracts of day 28 co-cultured 293T cells, which were used to generate the Q-PCR data shown in Figure S2A. Uninfected 293T cell lysates had a relatively high endogenous RT activity. Therefore, to help with the presentation of these data, this level was normalized to one and all of the other data were calculated relative to this value. The level of RT activity in PK-15 extracts was much higher than that of 293T cell extracts (+/−PERV) and it had reached saturation (out of range) when these data were collected.
(4.76 MB TIF)

Figure S3 Pig APOBEC3F Is Expressed in PK-15 Cells and its Over-expression Does Not Markedly Inhibit PERV Transmission. (A) An image of an ethidium bromide-stained agarose gel showing the results of an RT-PCR amplification experiment using PK-15 cellular RNA and appropriate controls. The top panel shows that PK-15 and representative PK-15 derived clones all expressed pig APOBEC3F, as indicated by the specific 175 bp pig APOBEC3F PCR product (confirmed by DNA sequencing). 293T cell mRNA and a diluted pig APOBEC3F expression plasmid were used as negative and positive controls, respectively. A larger, non-specific band was apparent only in the 293T cell RT-PCR reactions. The bottom panel shows that a conserved, 236 bp *beta-actin* gene fragment could be amplified from both PK-15 cells and human 293T cells (but not from diluted plasmid DNA). Note that this primer set differs from the human-specific set used in the Q-PCR experiments. The sizes of the marker (M) DNA bands are shown. (B) An image of an ethidium bromide-stained agarose gel showing expression of plasmid-derived pig APOBEC3F in PK-15 cells after 26 days of continuous co-culture. Non-transfected (NT) cells and diluted APOBEC3F plasmid DNA (pDNA) provided negative and positive controls, respectively. The larger 319 bp (far right lane only) and smaller 190 bp bands are the specific PCR products of the first and second rounds of semi-nested PCR, respectively (confirmed by DNA sequencing). (C) A histogram summarizing the level of PERV transmission that was observed after 23 days of co-culturing human 293T cells with PK-15 cells expressing a vector control or over-expressing pig APOBEC3F. Two datasets, each with an independent PK-15 clone in three replica co-culture wells, were collected in parallel and averaged for each histogram bar. One standard error of the mean is shown. The experimental parameters are identical to those used in Figure 1B.
(8.52 MB TIF)

Figure S4 Genetic Variation in Zoonosed PERV *pol* Gene Sequences. (A) Sequences of the PERV *pol* gene fragments cloned from 293T cells co-cultured with control vector-expressing PK-15 cells. The number of times that each sequence was recovered is shown (N). Experiments 1 and 2 used genomic DNA prepared from the 293T cells used to generate the data shown in Online Figure S2 (day 28 samples) and Figure 2B (day 23), respectively. The most frequently detected 147 bp PERV *pol* gene sequence is shown in its entirety (which together with PCR primers makes up the 193 bp product shown in Figure 5). Identical nucleotides in other sequences are represented by dashes and non-identical nucleotides by the indicated DNA bases. GenBank accession numbers are shown for *pol* gene fragments with 100% identity to previously reported sequences. (B) Sequences of the PERV *pol* gene fragments cloned from 293T cells co-cultured with control APOBEC3G-expressing PK-15 cells. Parameters are identical to those described above.
(0.05 MB DOC)
