# Supplementary figures and images for: The Restriction of Zoonotic PERV Transmission by Human APOBEC3G
Source: PLoS One. 2007 Sep 12;2(9):e893. doi: 10.1371/journal.pone.0000893 (PMC1963317; doi:10.1371/journal.pone.0000893)

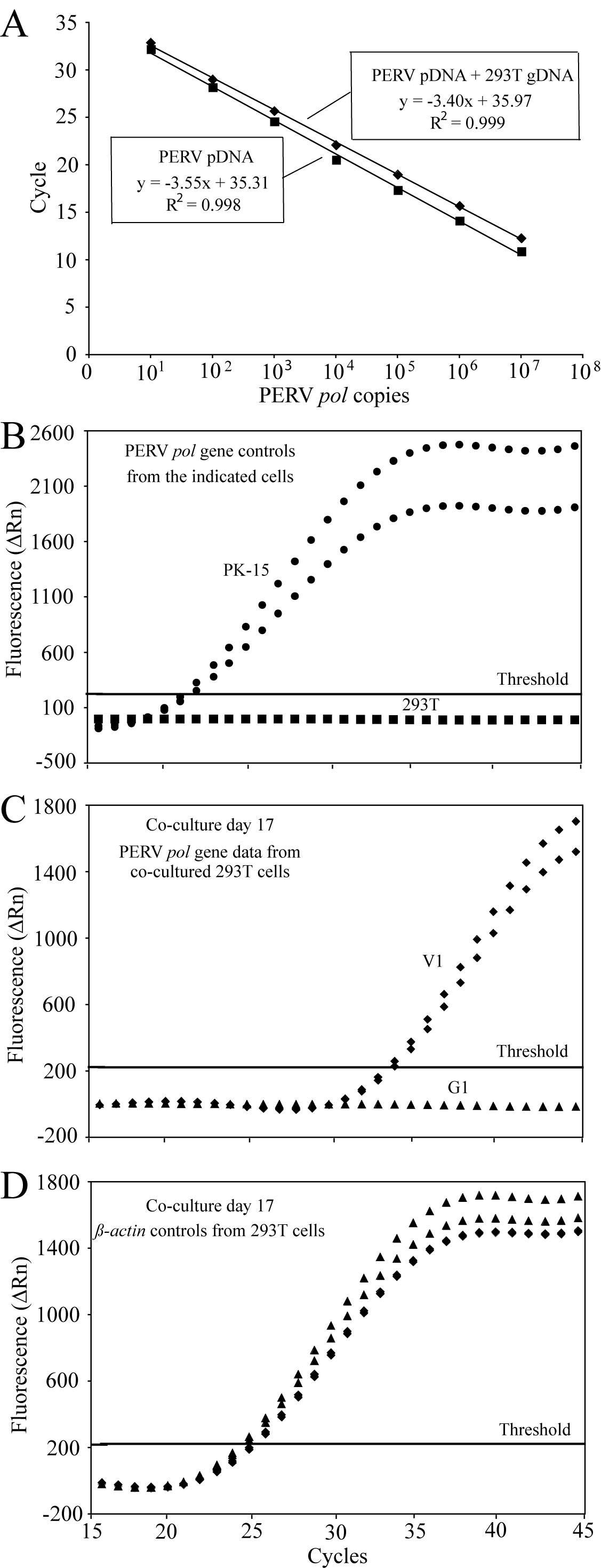

Supplement: Figure S1 — Quantitative Real-time PCR Analyses. (A) Standard curves depicting Q-PCR data obtained using dilutions of a linearized PERV pol gene plasmid alone (squares) or diluted plasmid plus 10 ng of 293T cell genomic DNA (diamonds). Under both conditions, all template amounts (10 to 107 copies) amplified efficiently (the log-linear slope equations are shown). The correlation co-efficiency value (R2), which reports the technical accuracy of the assay, is also indicated. The standard curve data points were the average of 2 independent reactions with deviations smaller than the symbols (i.e., CT errors for each point ranged from 0 to 0.4). (B) Two representative control Q-PCR datasets showing the amplification of PERV pol gene DNA from pig PK-15 cell genomic DNA (circles). Two additional control Q-PCR datasets showing that the PERV-specific primers fail to amplify product from uninfected human 293T cell genomic DNA (squares). The reaction threshold, 10 times the mean standard deviation of the background fluorescence level (BioRad), is indicated. (C) Representative co-culture Q-PCR amplification curves of PERV pol gene DNA. Template genomic DNA isolated from human 293T cells co-cultured with vector expressing PK-15 cells (diamonds) or human APOBEC3G-expressing PK-15 cells (triangles) was used. (D) Representative Q-PCR amplification curves of the 293T cell beta-actin gene, which served as an internal standard for quantifying the real-time PCR data. Raw Q-PCR data will be made available on request. (9.93 MB TIF) [file pone.0000893.s001.tif]

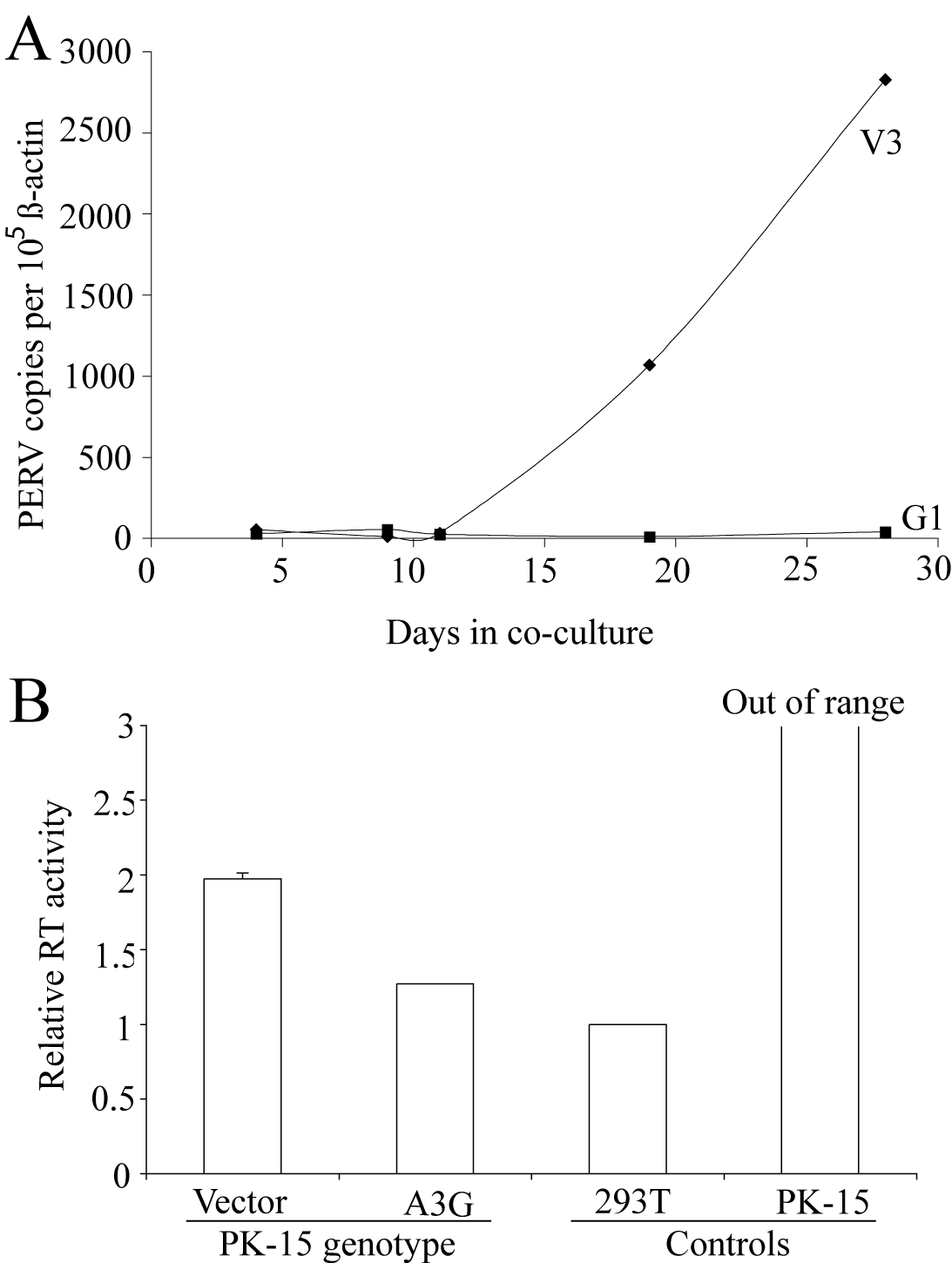

Supplement: Figure S2 — APOBEC3G inhibits PERV transmission. (A) A graph showing the accumulation of PERV pol gene-specific PCR products in 293T cells co-cultured with a control cell line (V3) but not with an APOBEC3G-expressing cell line (G1). The data points were an average of two Q-PCR runs and the difference between each run was smaller than the plotted symbol. The experimental parameters were identical to those used in the experiments shown in Figures 1B and 2B. (B) Relative levels of reverse transcriptase(RT)-activity detected in soluble extracts of day 28 co-cultured 293T cells, which were used to generate the Q-PCR data shown in Figure S2A. Uninfected 293T cell lysates had a relatively high endogenous RT activity. Therefore, to help with the presentation of these data, this level was normalized to one and all of the other data were calculated relative to this value. The level of RT activity in PK-15 extracts was much higher than that of 293T cell extracts (+/−PERV) and it had reached saturation (out of range) when these data were collected. (4.76 MB TIF) [file pone.0000893.s002.tif]

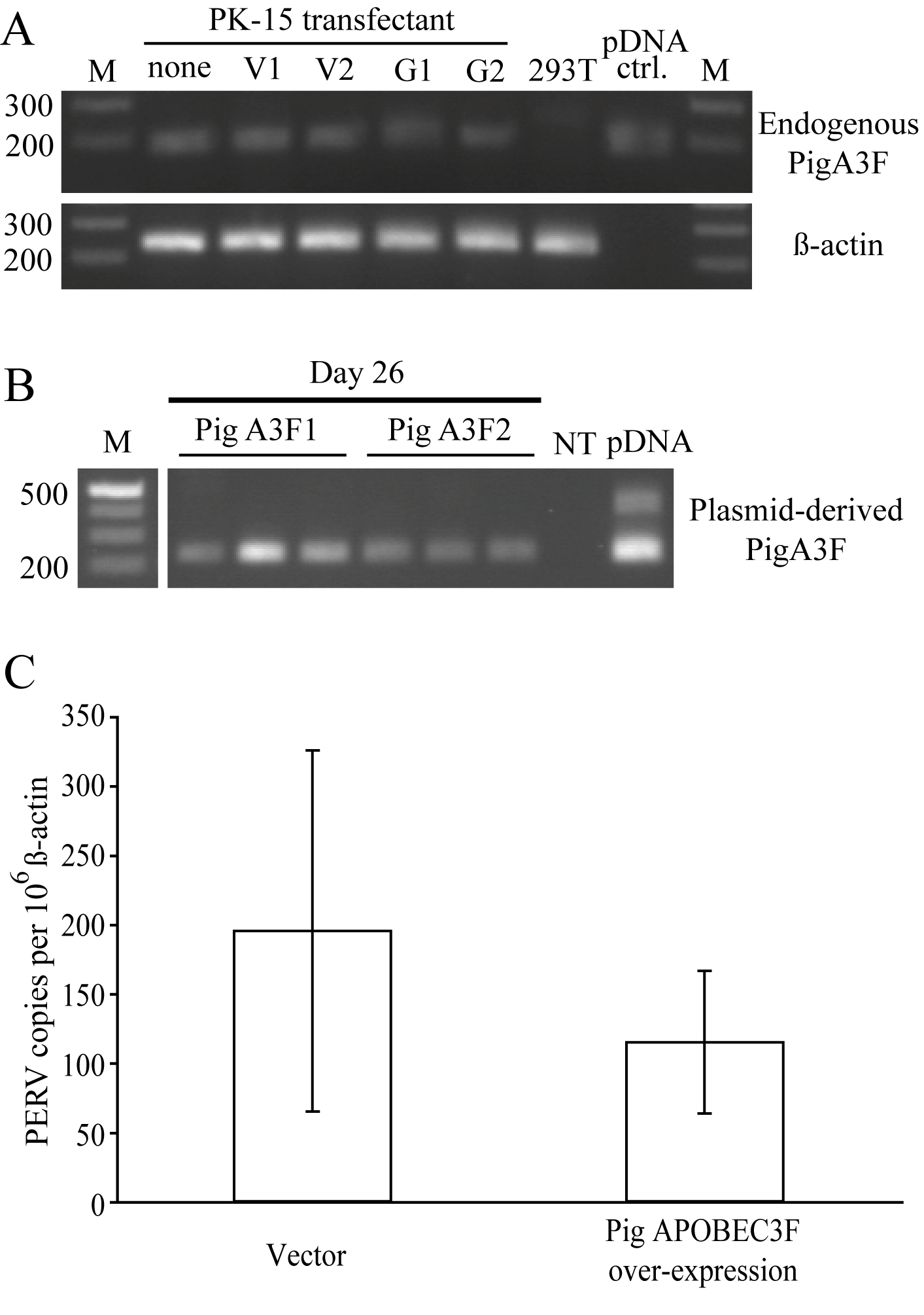

Supplement: Figure S3 — Pig APOBEC3F Is Expressed in PK-15 Cells and its Over-expression Does Not Markedly Inhibit PERV Transmission. (A) An image of an ethidium bromide-stained agarose gel showing the results of an RT-PCR amplification experiment using PK-15 cellular RNA and appropriate controls. The top panel shows that PK-15 and representative PK-15 derived clones all expressed pig APOBEC3F, as indicated by the specific 175 bp pig APOBEC3F PCR product (confirmed by DNA sequencing). 293T cell mRNA and a diluted pig APOBEC3F expression plasmid were used as negative and positive controls, respectively. A larger, non-specific band was apparent only in the 293T cell RT-PCR reactions. The bottom panel shows that a conserved, 236 bp beta-actin gene fragment could be amplified from both PK-15 cells and human 293T cells (but not from diluted plasmid DNA). Note that this primer set differs from the human-specific set used in the Q-PCR experiments. The sizes of the marker (M) DNA bands are shown. (B) An image of an ethidium bromide-stained agarose gel showing expression of plasmid-derived pig APOBEC3F in PK-15 cells after 26 days of continuous co-culture. Non-transfected (NT) cells and diluted APOBEC3F plasmid DNA (pDNA) provided negative and positive controls, respectively. The larger 319 bp (far right lane only) and smaller 190 bp bands are the specific PCR products of the first and second rounds of semi-nested PCR, respectively (confirmed by DNA sequencing). (C) A histogram summarizing the level of PERV transmission that was observed after 23 days of co-culturing human 293T cells with PK-15 cells expressing a vector control or over-expressing pig APOBEC3F. Two datasets, each with an independent PK-15 clone in three replica co-culture wells, were collected in parallel and averaged for each histogram bar. One standard error of the mean is shown. The experimental parameters are identical to those used in Figure 1B. (8.52 MB TIF) [file pone.0000893.s003.tif]
