# Supplementary material for: The Restriction of Zoonotic PERV Transmission by Human APOBEC3G
Source: PLoS One. 2007 Sep 12;2(9):e893. doi: 10.1371/journal.pone.0000893 (PMC1963317; doi:10.1371/journal.pone.0000893)
Supplement: Figure S4 — Genetic Variation in Zoonosed PERV pol Gene Sequences. (A) Sequences of the PERV pol gene fragments cloned from 293T cells co-cultured with control vector-expressing PK-15 cells. The number of times that each sequence was recovered is shown (N). Experiments 1 and 2 used genomic DNA prepared from the 293T cells used to generate the data shown in Online Figure S2 (day 28 samples) and Figure 2B (day 23), respectively. The most frequently detected 147 bp PERV pol gene sequence is shown in its entirety (which together with PCR primers makes up the 193 bp product shown in Figure 5). Identical nucleotides in other sequences are represented by dashes and non-identical nucleotides by the indicated DNA bases. GenBank accession numbers are shown for pol gene fragments with 100% identity to previously reported sequences. (B) Sequences of the PERV pol gene fragments cloned from 293T cells co-cultured with control APOBEC3G-expressing PK-15 cells. Parameters are identical to those described above. (0.05 MB DOC) [file pone.0000893.s004.doc]

**A** PERV transfers in vector control experiments

N Expt Sequence GB #

5 1 GACCAGTATGGCCACAGCTGCGATAGCCTTCAGACATACGGGCCAACCACTGGCTACAGGATCAAGCTTCTTTGACAGGTAGGCAACAGGTCTCCTCCATGGTCCTAGGGTTTGGGTTAAAACTCCTCGGGCTACTCCCTTACGCTC AY099323

4 1 ---------------------------------G-----------------------------G--------------------------------------------A-----------------C-------------------- AF435967

3 2 ---------------------------------G-----------------------------G--------------------------------------------------------------C--------------------

2 1 ---------------T-----------------G-----------------------------G--------------------------------------------------------------C--------C--------T--

1 1 ---------------------------------G-----------------------------------------------------------------------------------------------------------------

1 1 ------C--------------------------G----T------T-----------------G-------------A---------------T--------------------------------C-----C--------------

1 1 ---------------------------------G-----------------------------G-------------------------------------------A------------------C--------------------

1 1 ---------------------------------G-----------------------------G--------------------------------------------A-------------T---C--------------------

1 1 ---------------------------------G-----------------------------G--------------------------------------------------------------C----------T---------

1 1 ---------------------------------G----T------------------------G--------------------------------------------A-----------------C--------------------

1 1 ---------------------------------G-----------G-----------------G--------------------------------------------------------------C----------------AT--

1 2 ------C--------------------------G----T------T-----------------G-------------A---------------T--------------------------------C-----C--------------

**B** PERV transfers in human APOBEC3G experiments

N Expt Sequence GB #

17 1 GACCAGTATGGCCACAGCTGCGATAGCCTTCAGACATACGGGCCAACCACTGGCTACAGGATCAAGCTTCTTTGACAGGTAGGCAACAGGTCTCCTCCATGGTCCTAGGGTTTGGGTTAAAACTCCTCGGGCTACTCCCTTACGCTC AY099323

8 2 --------------------------------------------------------------------------------------------------------------------------------------------------- AY099323

3 1 ---------------------------------G-----------------------------------------------------------------------------------------------------------------

1 1 -----------------------------------------A---------------------------------------------------------------------------------------------------------
